# Quantification of myocardial infarct area based on T_RAFFn_ relaxation time maps - comparison with cardiovascular magnetic resonance late gadolinium enhancement, T_1ρ_ and T_2_ in vivo

**DOI:** 10.1186/s12968-018-0463-x

**Published:** 2018-06-07

**Authors:** Elias Yla-Herttuala, Svetlana Laidinen, Hanne Laakso, Timo Liimatainen

**Affiliations:** 1 A.I. Virtanen Institute for Molecular Sciences, University of Eastern Finland, Kuopio, Finland; 2 Center for Magnetic Resonance Research, Minneapolis, MN USA; 3 Research Unit of Medical Imaging, Physics and Technology, University of Oulu, Oulu, Finland; 4 Department of Diagnostic Radiology, University Hospital of Oulu, P.O. Box 50, 90029 OYS Oulu, Finland

**Keywords:** Cardiovascular magnetic resonance, Magnetic resonance imaging (MRI), Myocardial infarction (MI), Relaxation time, T_RAFF2_, T_RAFF4_, T_1ρ_, T_2_, LGE, Sirius red staining

## Abstract

**Background:**

Two days after myocardial infarction (MI), the infarct consists mostly on necrotic tissue, and the myocardium is transformed through granulation tissue to scar in two weeks after the onset of ischemia in mice. In the current work, we determined and optimized cardiovascular magnetic resonance (CMR) methods for the detection of MI size during the scar formation without contrast agents in mice.

**Methods:**

We characterized MI and remote areas with rotating frame relaxation time mapping including relaxation along fictitious field in n^th^ rotating frame (RAFFn), T_1ρ_ and T_2_ relaxation time mappings at 1, 3, 7, and 21 days after MI. These results were compared to late gadolinium enhancement (LGE) and Sirius Red-stained histology sections, which were obtained at day 21 after MI.

**Results:**

All relaxation time maps showed significant differences in relaxation time between the MI and remote area. Areas of increased signal intensities after gadolinium injection and areas with increased T_RAFF2_ relaxation time were highly correlated with the MI area determined from Sirius Red-stained histology sections (LGE: R^2^ = 0.92, *P* < 0.01, T_RAFF2_: R^2^ = 0.95, *P* < 0.001). Infarct area determined based on T_1ρ_ relaxation time correlated highly with Sirius Red histology sections (R^2^ = 0.97, *P* < 0.01). The smallest overestimation of the LGE-defined MI area was obtained for T_RAFF2_ (5.6 ± 4.2%) while for T_1ρ_ overestimation percentage was > 9% depending on T_1ρ_ pulse power.

**Conclusion:**

T_1ρ_ and T_RAFF2_ relaxation time maps can be used to determine accurately MI area at various time points in the mouse heart. Determination of MI size based on T_RAFF2_ relaxation time maps could be performed without contrast agents, unlike LGE, and with lower specific absorption rate compared to on-resonance T_1ρ_ relaxation time mapping.

## Background

Cardiovascular diseases are the leading causes of death worldwide [1, 2]. Myocardial infarction (MI) is caused by a complete or partial blockage of the coronary artery, leading to inflammation, arrhythmia, and prolonged absence of perfusion [3–8]. The formation of fibrosis and collagen together with the loss of myocytes can lead to harmful remodeling of the myocardium and finally heart failure [3–7, 9]. Perfusion deficits cause cell death via necrosis and increases in extracellular space, which increases the tissue free water content and affects water-macromolecular interactions [10]. MI is a dynamic process since further loss of myocytes may occur, and collateral angiogenesis may decrease the infarct volume as a function of time [4, 6]. Scar tissue eventually replaces the damaged myocytes within 1–2 weeks after MI [4].

Several cardiovascular magnetic resonance (CMR) methods, for example T_2_ and T_1ρ_ relaxation time mappings and CMR spectroscopy, have been implemented to detect both acute and chronic MI [11–13]. Currently, the golden standard to detect chronic or irreversible injury using CMR is late gadolinium (Gd) enhancement (LGE). LGE creates a high contrast between normal myocardium and irreversible infarcted areas [11, 14, 15]. Contraindications for the use of Gd-based contrast agents are known Gd allergy and acute or chronic renal dysfunction [11, 16], which limit its clinical use.

Conventional transverse, or spin-spin, relaxation time, T_2_, shows the difference between acute and chronic MI [15, 17]. Edema in the acute infarct phase increases free water content, significantly affecting heart function and T_2_ relaxation time [12, 18]. Regions of acute MI can involve a mixture of tissue edema, hemorrhage, and inflammation, which leads to the underestimation of water movement in extracellular space and the overestimation of the infarct area in the T_2_ relaxation time map [15, 19].

Rotating frame relaxation times are used to characterize the relaxation during radiofrequency (RF) pulses. This differs from conventional T_1_ and T_2_ relaxations where relaxation occurs during free precession. Longitudinal rotating frame relaxation time, T_1ρ_, refers to relaxation along the RF field, which takes place typically during on-resonance RF irradiation. When RF irradiation is on-resonance and spins are locked along the RF field, the spins experience the RF field, instead of the main magnetic field. This leads to sensitivity of T_1ρ_ to slow molecular motions with frequencies close to RF pulse frequency, which are typically in the range of 0.1 to 10 kHz in vivo*.* Comparatively, conventional T_1_ is sensitive for Larmor frequency of the main magnetic field (B_0_) which is typically in the range 10–500 MHz resembling high frequencies, i.e., fast molecular motions including the motion of free water. Increased T_1ρ_ relaxation in MI has been associated with increased extracellular volume and alterations in proton exchange between water and macromolecules [11, 16]. Furthermore, T_1ρ_ relaxation times are affected by macromolecule concentrations, viscosity, molecular weight and pH, since these factors change water mobility in tissue. Collectively, these factors affect molecular correlation times and therefore can explain T_1ρ_ relaxation time increases in MI and other pathologies [16, 20]. Area with elevated T_1ρ_ relaxation time show high correspondence with the MI area detected by LGE in mice [21] and in humans [11].

Specific absorption rate (SAR) often limits rotating frame relaxation measurements, especially in clinical settings, since high SAR may lead to tissue heating. One method to reduce SAR in rotating frame relaxation measurements is relaxation along a fictitious field (RAFF) in n^th^ rotating frame (RAFFn) [22–24]. RAFFn is produced by nested sine amplitude and cosine frequency modulated RF pulses operating in a sub-adiabatic regime and RF waveforms become more complicated when n increases [22, 23]. A fast, sub-adiabatic sweep of the effective RF field produces a fictitious field, which forms a part of the final effective RF field and magnetization precesses around this effective field [22]. When n increases in RAFFn, the tolerance for B_0_ and radiofrequency field (B_1_) inhomogeneities increases [23]. Due to lower flip angles with increasing n, the pulse bandwidth increases significantly [24]. Amplitude and frequency modulations, increase of bandwidth, decrease of flip angle together with remarkably lower (approximately 80%) SAR-values of RAFF4 and (approximately 30%) SAR-values of RAFF2 compared to continuous wave spin lock (T_1ρ_) are clear advantages of RAFFn [22–24] and make RAFFn more suitable for clinical use than T_1ρ_.

In the current study, we have optimized infarct sizing using T_RAFF2_ and T_RAFF4_ relaxation time mappings. The results were compared with T_1ρ_ and T_2_ relaxation time mappings, LGE and histology staining with Sirius Red.

## Methods

### Animal model

The left anterior descending artery (LAD) was ligated permanently in 10 female C57BL mice (20-24 g) as previously described [25]. Mice were anesthetized by 4% of isoflurane (Piramal Healthcare, Northumberland, UK), and anesthesia was maintained with 2.0% during the operation. The left side of mouse chest from sternum to Linea axillaris posterior was shaved and disinfected with 75% ethanol. An approximately 1.5 cm long transversal incision was made at the level of the fourth rib to left intercostal space. Through the incision and with help of a self-retaining retractor, the heart was exposed. The LAD was ligated with a 6.0 silk suture approximately at midway between its origin and the apex of the heart. After the LAD ligation, the heart was placed back to its original location. The skin were sutured in layers with 5.0 nylon suture. After the surgery, 0.05–0.1 mg/kg buprenorphine (0.3 mg/ml Temgesic, RB Pharmaceuticals, Slough, UK) and 5 mg/kg carprofen (50 mg/ml, Rimadyl, Pfizer Oy Animal Health, Helsinki, Finland) for analgesia were injected subcutaneously and repeated at days 1 and 2 after the surgery. All surgical procedures were performed according to protocols approved by the Finnish Committee for the use and care of laboratory animals.

### CMR

Mice underwent CMR at 1 (*n* = 10), 3 (*n* = 9), 7 (*n* = 5) and 21 (*n* = 5) days after LAD occlusion. All experiments were performed using a horizontal 9.4 T magnet (Varian Inc. Palo Alto, California, USA) with a gradient set with maximum gradient strength of 600 mT/m and controlled by a Bruker console (Bruker GmbH, Ettlingen, Germany). Quadrature volume transceiver with a coil diameter of 35 mm (Rapid Biomed GmbH, Ettlingen, Germany) was used for all CMR experiments. Mice were anesthetized for CMR with 4% isoflurane mixed in oxygen and nitrogen with ratio of 1:3. The level of isoflurane was decreased to 1% for the imaging. Mouse body temperature was kept close to 37 °C by circulating warm water tubes placed under the mouse. Electrocardiography (ECG) was measured from fore paws using needle electrodes and a pneumatic pillow placed under the mouse monitored respiration. Both signals were registered using Model 1025 monitoring and gating system (Small Animal Instruments Inc., Stony Brook, New York, USA) during the experiments. Both ECG and respiration signals were used to gate CMR experiments.

Multi-slice cine images covering the whole heart were taken using fast imaging with balanced steady state precession (FISP) readout sequence. The imaging parameters for cine images were FOV = 4 × 4 cm^2^, slice thickness = 1 mm, matrix size = 192 × 192, TE = 1.9 ms, TR = 8.0 ms, scan TR = 99.0 ms, flip angle = 10° and number of frames 10–11 depending on mouse heart rate. Depending on the size of the heart, 8–10 slices were imaged.

**Fig. 1:**
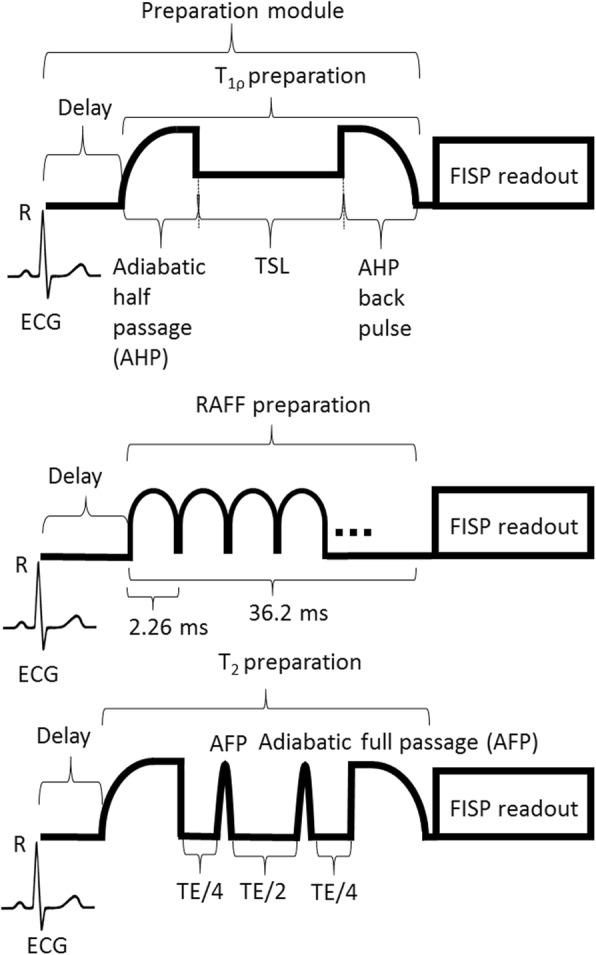
An illustration of CMR pulse sequences consisting of the preparation module for different weightings and FISP readout. ECG, electrocardiogram;  TE, echo time

The rotating frame preparation modules used to measure T_RAFFn_ consisted of RAFF2 or RAFF4 pulses (pulses RF power (γB_1_/(2π)) 1250 Hz and 648 Hz, respectively, duration 2.26 ms) which were applied in pulse trains of lengths 0, 9.1, 18.1 and 36.2 ms. Before the RAFFn pulse train, a delay with durations of 36.2, 27.15, 18.1 and 0 ms, respectively, was added to adjust imaging to occur at the same cardiac phase for weightings with different durations. An illustration of a rotating frame preparation module and readout sequence is shown in Fig. 1.

T_1ρ_ preparation was performed using a rotating frame preparation module (Fig. 1) which contained adiabatic half passage (AHP) pulse (power 1250 Hz, duration 2.0 ms), continuous wave spin-lock-pulse with time-to-spin-lock (TSL) = 0.4, 9.4, 27.4 and 45.4 ms and AHP-back pulse (power 1250 Hz, duration 2.0 ms) [21]. Before T_1ρ_ preparation a delay (45.4, 27.4, 9.4 and 0 ms, respectively to TSL) was added. T_1ρ_ dispersion was measured by altering the spin lock power (γB_1_/(2π)) in a range of (400, 625 and 1250 Hz) and keeping AHP and AHP-back pulses the same.

T_2_ measurements were conducted using Hahn double echo preparation containing an AHP excitation-pulse (power 1250 Hz, duration 3.0 ms), two Hyperbolic Secant (HS1)-pulses (power 1250 Hz, duration 4.5 ms) and a reversed AHP-pulse (power 1250 Hz, duration 3.0 ms) (Fig. 1). Between the pulses symmetric delays were used resulting in total TEs of 0.05, 2.3, 4.5, and 14.0 ms. Delays in front of T_2_ preparation were 14.0, 4.5, 2.3 and 0.05 ms, respectively.

B_1_ was measured by applying a block pulse with power of 625 Hz. The B_1_ block pulse was applied with pulse durations 0, 0.25, 0.5, 0.75, 1.0, 1.25, 1.5 and 1.75 ms [26].

All relaxation time maps and B_1_ measurements were acquired using a FISP-readout sequence in a single short-axis slice at the mid-ventricular level. The following parameters: FOV = 4 × 4 cm^2^, slice thickness = 1 mm, matrix size = 256 × 256 (for B_1_ measurements, the matrix size was 128 × 128), TE = 1.9 ms, TR = 14.9 ms, and flip angle = 90° were used for the FISP-readout. A delay between weighting pulses depended on respiratory rate being at least 1460 ms.

At the last time point before sacrificing the mice, LGE images were acquired in the same slice as all other measurements using an inversion prepared pulse sequence with an inversion time of 300 ms, FISP-readout, FOV = 4 × 4 cm^2^, slice thickness = 1 mm, matrix size = 256 × 192, TE = 2.0 ms, TR = 5.6 ms, scan TR = 3000.0 ms and flip angle = 90° [27]. The gadobutrol (Gadovist, Bayern Oy, Turku, Finland) intravenous injection volume was 5 ml/kg per mouse.

Only five mice survived to 21 days and were sacrificed for histology after imaging. For histology, the hearts were perfused through the left ventricle with phosphate buffered saline and then immersion fixed with 4% paraformaldehyde with sucrose in phosphate buffered solution for 4 h. After 4 h, the hearts were placed into 15% sucrose. Paraffin-embedded, 4 μm thick, cross-sections of the heart were stained with Sirius Red to determine the fibrotic areas of the infarcted myocardium. Histological sections were analyzed and photographed with microscopy (Nikon Eclipse, Ni-E, Tokyo, Japan).

### Data analysis

All relaxation time maps were reconstructed from signal intensities with pixel-by-pixel analysis using Aedes software package (http://aedes.uef.fi/) in Matlab platform (Mathworks Inc. Natick, Massachusetts, USA). T_1ρ_ and T_2_ relaxation time maps were fitted using linear function for linearized data. T_RAFF2_ and T_RAFF4_ were fitted by using single mono-exponential decay function without taking into account the steady state formation. Regions of interest (ROIs) were manually traced with visual delineations of MI and remote areas based on relaxation time maps, cine images and images of Sirius Red-stained sections. End systolic volume (ESV), end diastolic volume (EDV), ejection fraction (EF), and cardiac output (CO) were defined based on endocardial border in cine images.

Infarct percentage analysis was done with midline length-based method with a function of (L_(infarct)_/ L_(circumference)_)•100%, where L denotes measured length from either T_RAFF2_, T_RAFF4_, T_1ρ_, T_2_, LGE or Sirius Red-stained section [28]. Relative relaxation time difference (RRTD) values were calculated with function of (T_(infarct)_ –T_(remote)_)/ T_(remote)_, where T denotes either T_RAFF2_, T_RAFF4_, T_1ρ_ or T_2_ relaxation time.

Amount of overestimation (AOE) of infarct area relative to the LGE-defined MI area, as the gold standard, was calculated based on midline length-based method with a function of ((L_(infarct)_ –LGE_(infarct)_)/ L_(infarct)_) •100%, where L denotes either T_RAFF2_, T_RAFF4_, T_1ρ_ or T_2_ relaxation time [29].

Statistics: All numerical values are given as mean ± standard deviation (SD). Two-way ANOVA with Bonferroni post hoc testing was applied to compare the spatial and temporal changes between the infarct and remote areas of myocardium, and the analyses were performed using GraphPad Prism software (GraphPad Software, La Jolla, California, USA). Two-way ANOVA with Bonferroni post hoc testing was performed to compare changes between RRTD values of different relaxation times. One-way ANOVA with Bonferroni post hoc testing for multiple comparisons were applied to compare time point differences between relaxation times and also the differences between time points of cardiac functions.

## Results

**Fig. 2:**
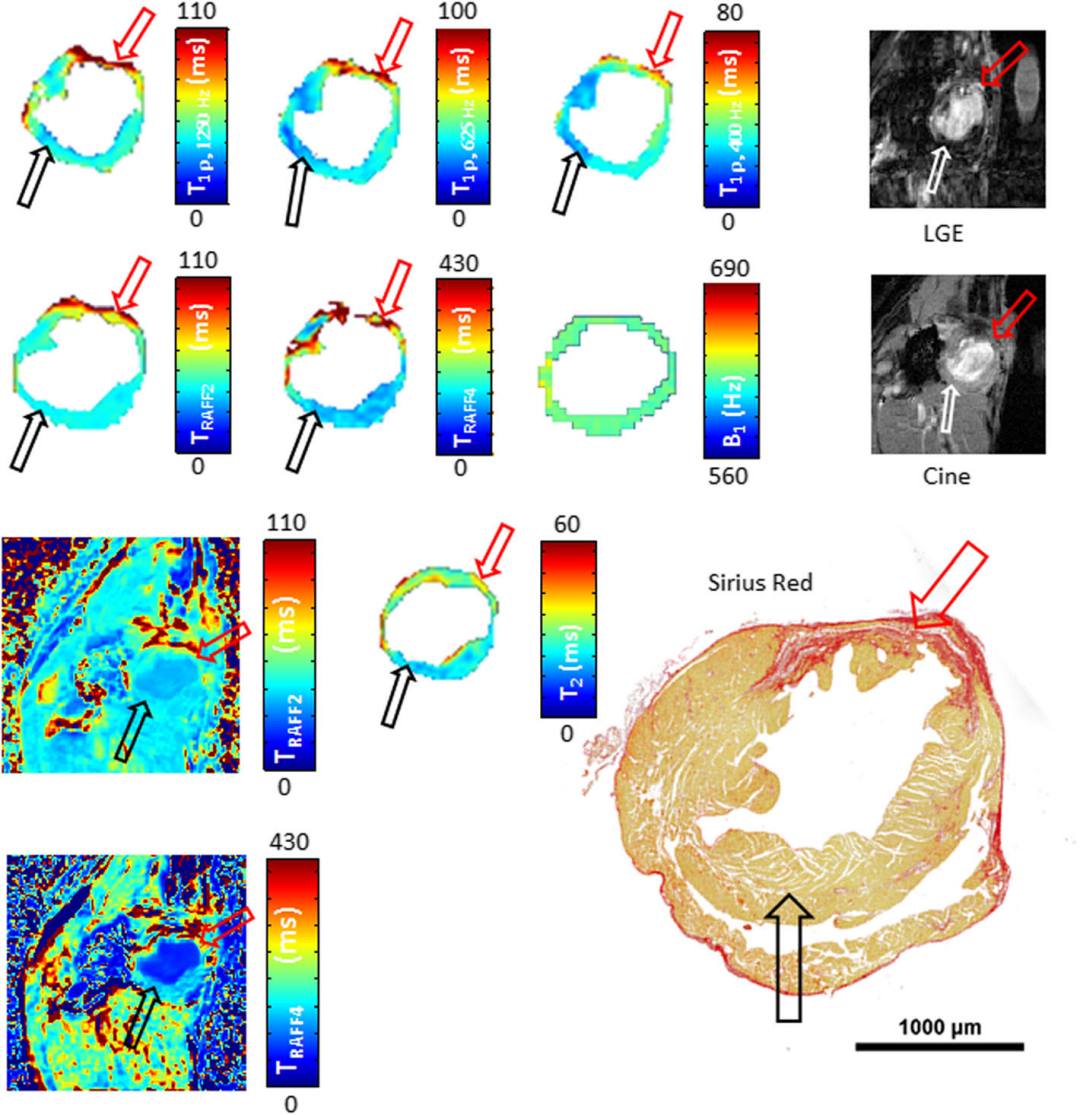
Relaxation time maps, late gadolinium enhancement (LGE), cine and a corresponding histology image with Sirius Red-stained section from infarcted mouse heart at the last (21 day) time point after left anterior descending (LAD) myocardial infarction (MI). Red arrows indicate the infarct area and black/white arrows show the remote control area. B_1_ homogeneity was verified to be nominal ±10% Hz in the area of the whole myocardium

Increased relaxation time constants were found in the MI areas after LAD ligation. Infarct areas obtained with relaxation time mappings were compared with infarct areas derived based on LGE-images, cine-images and Sirius Red-stained histological sections (Fig. 2).

**Fig. 3:**
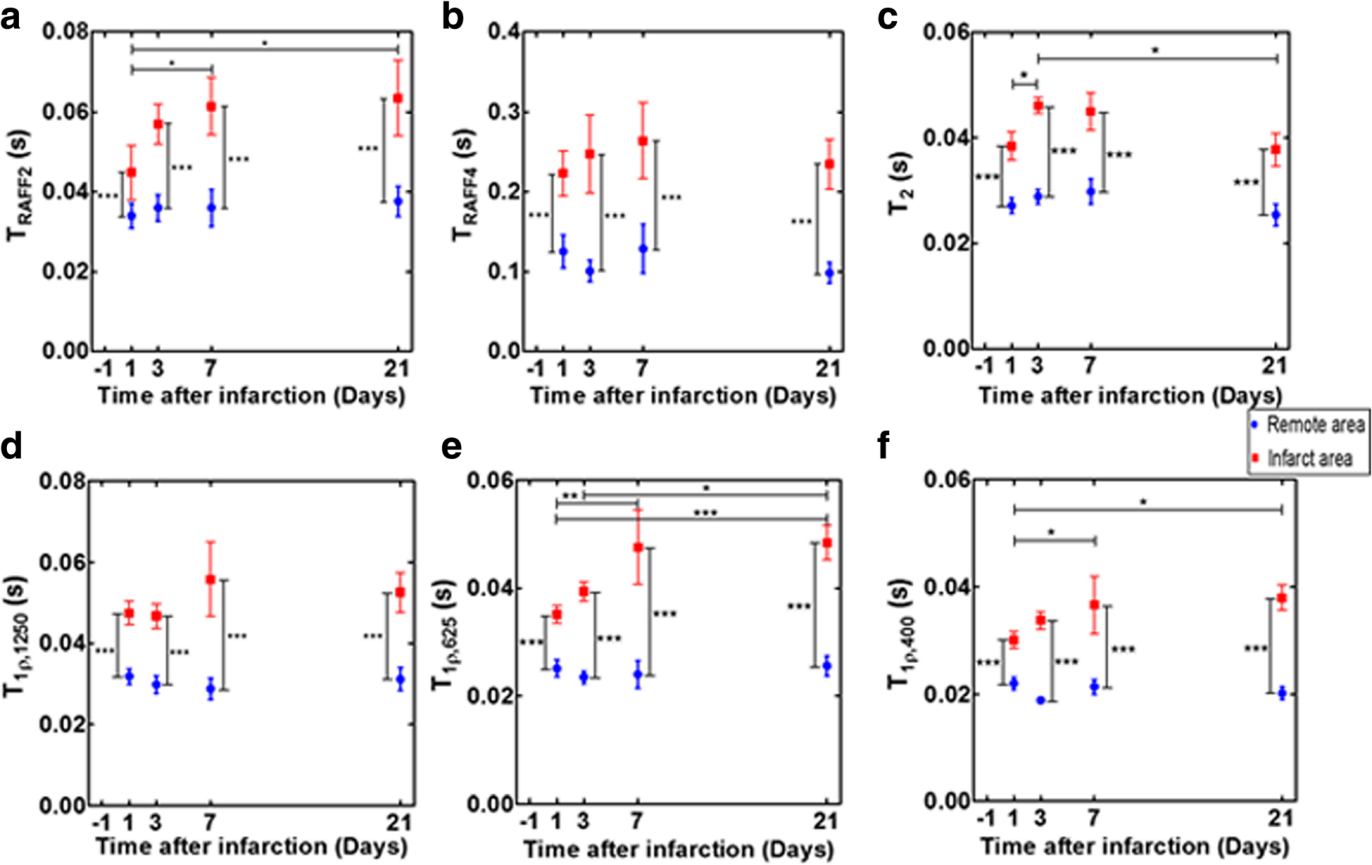
Different relaxation times (mean ± SD). T_RAFF2_ (**a**), T_RAFF4_ (**b**), T_2_ (**c**), T_1ρ1250_ (**d**), T_1ρ625_ (**e**) and T_1ρ400_ (**f**) were calculated in infarct and remote areas at different time points after the LAD ligation. Blue circles indicate remote and red squares infarct area. (*P < 0.05, **P < 0.01 and ***P < 0.001, Two-way ANOVA with Bonferroni post hoc test)

T_RAFF2_ relaxation times were significantly higher in the infarct areas compared to remote areas (*P* < 0.001) (Fig. 3a). T_RAFF2_ relaxation times in infarct area increased significantly up to 7 days and remained elevated until day 21 after the LAD ligation (*P* < 0.05, *P* < 0.05, respectively) (Fig. 3a). T_RAFF4_ relaxation time in the infarct area was significantly elevated at all time points compared to the remote area (*P* < 0.001, Fig. 3b). The remaining relaxation times (T_1ρ1250_, T_1ρ625_, T_1ρ400_ and T_2_) were significantly elevated in the infarct area compared to remote areas (*P* < 0.001, respectively), and there were significant differences, except in T_1ρ1250_, between time points (*P* < 0.05, respectively, Fig. 3c-f). Specifically, T_1ρ625_ relaxation times increased significantly in the infarct area at 7 days after the LAD ligation (*P* < 0.01) and remained elevated until day 21 (*P* < 0.001, Fig. 3e). There was a significant increase in T_1ρ625_ relaxation times in infarct area at days 7 and 21 compared to day 1 after the LAD occlusion (*P* < 0.05, *P* < 0.05 respectively), and it also increased significantly from day 3 to day 21 (*P* < 0.05, Fig. 3e). Additionally, the trend between infarct and remote areas in T_1ρ625_, T_1ρ400_ and T_2_ relaxation times differed significantly (*P* < 0.05, respectively) from each other (Fig. 3c, e, f). A decrease in T_2_ relaxation time was detected from day 3 to day 21 after the LAD ligation (*P* < 0.05, Fig. 3c).

There were no significant changes in the relaxation times in the remote areas between the imaging time points (*P* > 0.05, Fig. 3).

RRTD provided a measure of differences in relaxation time values between infarct and remote areas for the relaxation measurements (Table 1). RRTD values of T_RAFF4_ differed significantly from other relaxation time methods at several different time points (Table 1). There were significant differences in RRTD values of some of the T_1ρ_ relaxation times when comparing RRTD values at day 1 (Table 1).

**Table 1:** Relative relaxation time difference (RRTD) values formed by infarct and remote area relaxation times presented as mean ± SD

Relaxation time constant	Day 1	Day 3	Day 7	Day 21
T_1ρ1250_	0.49 ± 0.11^□^	0.60 ± 0.29^□□□^	0.91 ± 0.42*	0.69 ± 0.16^□□^
T_1ρ625_	0.41 ± 0.09^□□^	0.70 ± 0.27^□□□^	0.99 ± 0.44**	0.90 ± 0.24*
T_1ρ400_	0.37 ± 0.09^□□^	0.79 ± 0.21**,^□□^	0.69 ± 0.33^□^	0.90 ± 0.27**
T_2_	0.41 ± 0.11^□□^	0.61 ± 0.19^□□□^	0.51 ± 0.11^□□^	0.50 ± 0.19^□□□^
T_RAFF2_	0.41 ± 0.12^□□^	0.62 ± 0.43^□□□^	0.73 ± 0.22	0.66 ± 0.21^□□^
T_RAFF4_	0.90 ± 0.45	1.36 ± 0.62	1.22 ± 0.59	1.39 ± 0.37

Significance of differences in RRTD values were calculated by Two-way ANOVA with Bonferroni post hoc test (^□^=*P*<0.05, ^□□^=*P*<0.01, ^□□□^=*P*<0.001 for difference to T_RAFF4_ at that specific time point) and One-way ANOVA with Bonferroni post hoc test (*=*P*<0.05, **=*P*<0.01 for difference to day 1)

**Fig. 4:**
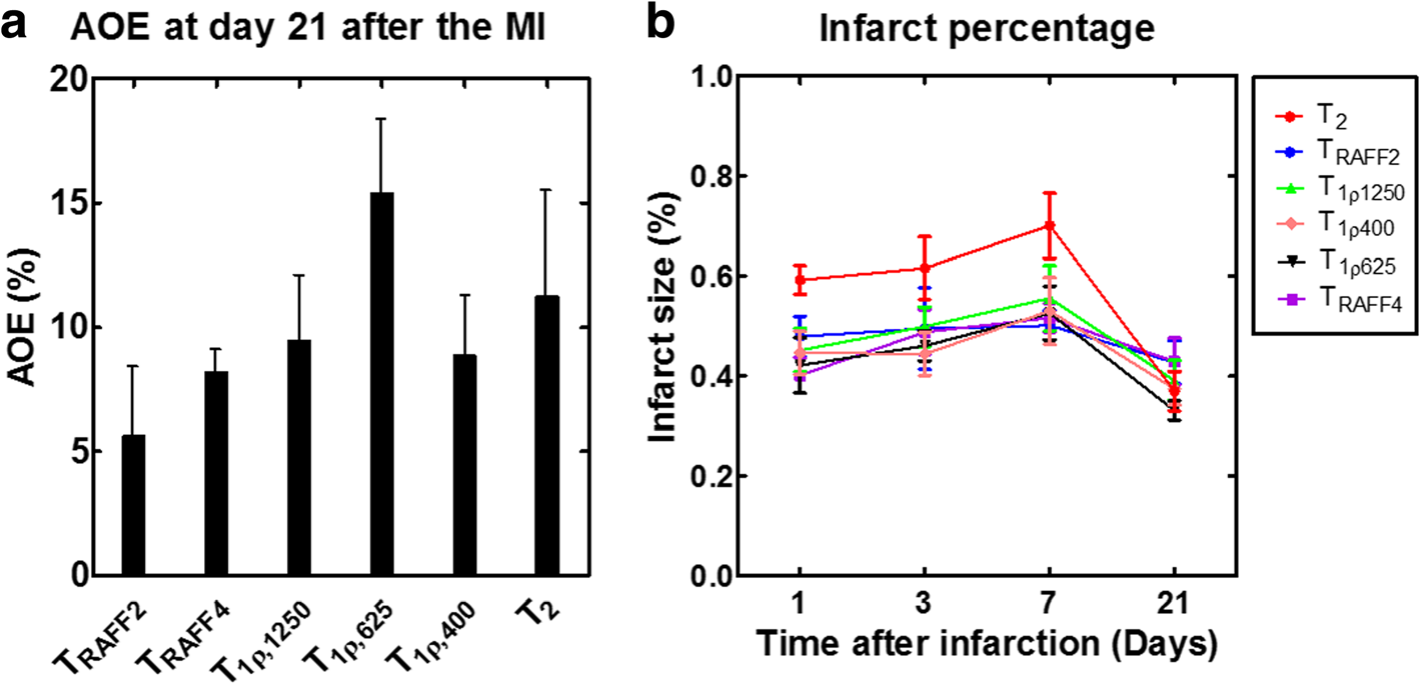
Amount of overestimation (AOE) with regard to the LGE-defined MI area based on the relaxation time maps at 21 days after infarct (**a**) and infarct percentages at every time point based on the relaxation time maps (**b**)

AOE values were determined for all relaxation times (Fig. 4a). AOE was lowest for T_RAFF2_ indicating that area of increased T_RAFF2_ is most similar to LGE-measured infarct area (Fig. 4a).

**Fig. 5:**
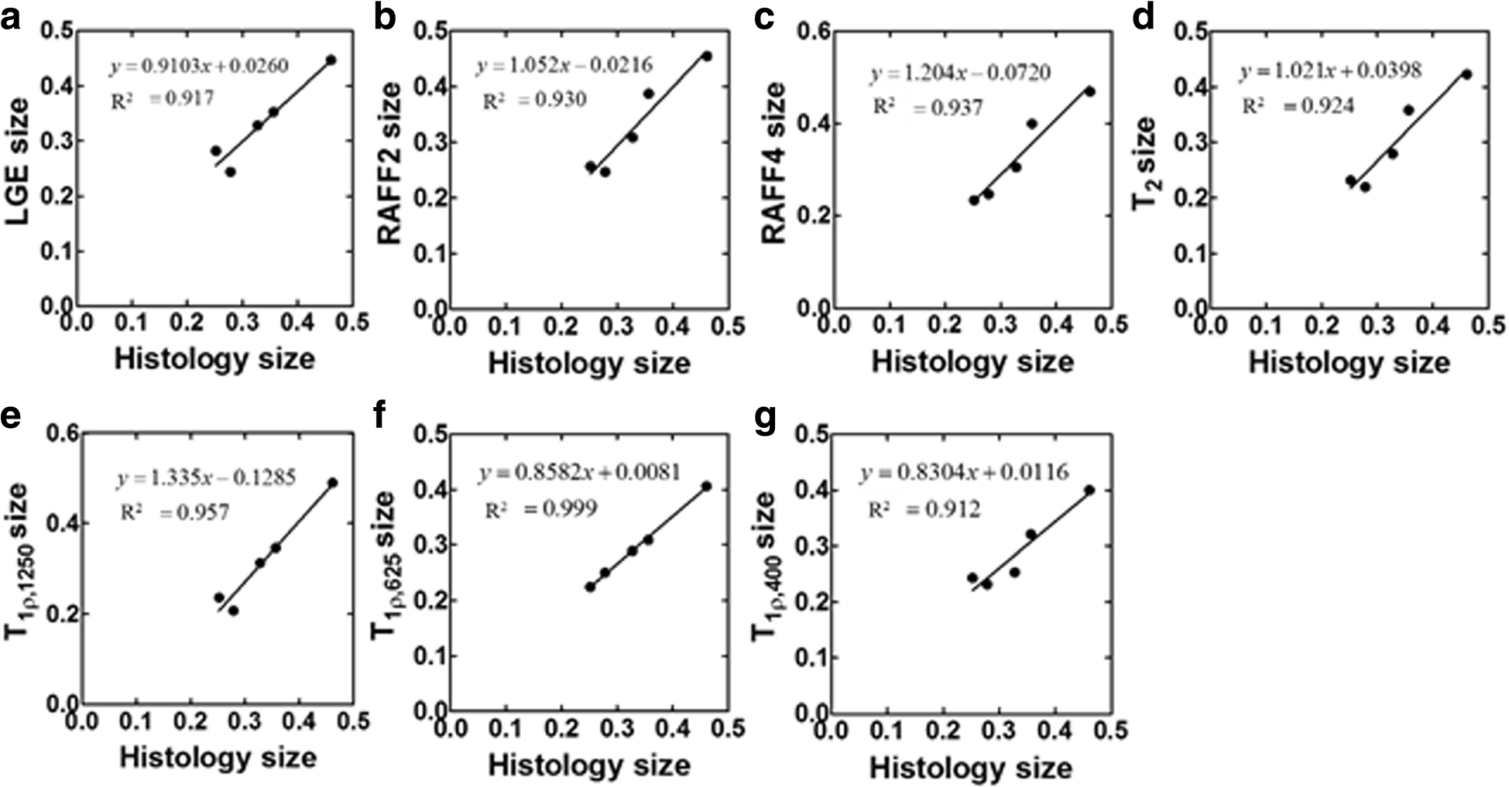
Linear correlation from the ratios between the arc of infarct and the circumferences of the whole myocardium determined from LGE-image (**a**), T_RAFF2_ (**b**), T_RAFF4_ (**c**), T_2_ (**d**), T_1ρ1250_ (**e**), T_1ρ625_ (**f**), T_1ρ400_ (**g**) and Sirius Red-stained histology sections*.* Formulas of linear relationships are shown next to correlation line

Infarct sizes were calculated also as a ratio between the arc of infarct and the circumference of the whole myocardium from relaxation time maps and LGE-images (Figs. 4b and 5). These infarct sizes were correlated with the ones measured based on Sirius Red-stained histology-images (Fig. 5). The highest Spearman correlations were obtained with T_1ρ_ (R^2^ = 0.97, *P* < 0.01). Infarct sizes from T_RAFF2_ (R^2^ = 0.93, *P* < 0.001) and T_RAFF4_ (R^2^ = 0.94, *P* < 0.001) showed a high correlation as well as LGE (R^2^ = 0.92, *P* < 0.01) with infarct size from Sirius Red-stained sections (Fig. 5). The infarct fraction given as a percentage at early time points obtained from T_2_ relaxation time map was largest but decreased to similar percentages as obtained from the other relaxation time maps at day 21 (Fig. 4b).

**Fig. 6:**
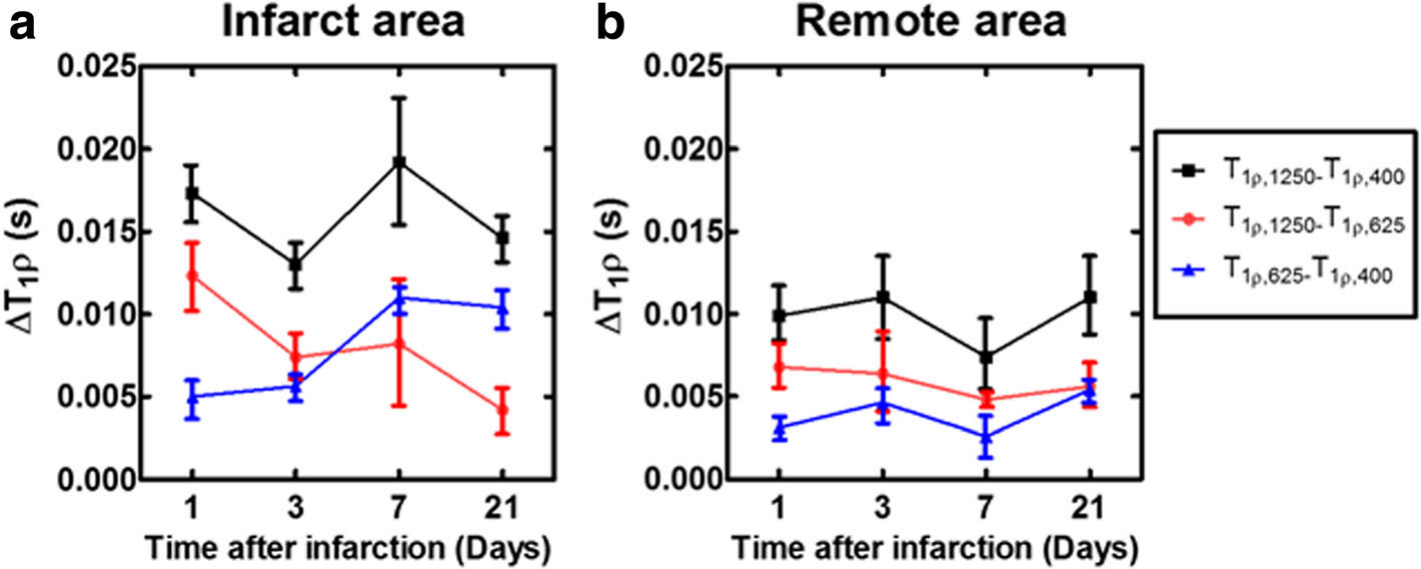
Dispersion of T_1ρ_ relaxation time in infarct area (**a**) and in remote area (**b**). Red color shows subtraction between T_1ρ1250_ - T_1ρ625_, black color shows subtraction between T_1ρ1250_ - T_1ρ400_ and blue color shows subtraction between T_1ρ625_ - T_1ρ400_

Dispersion of T_1ρ_ relaxation times (ΔT_1ρ_) was calculated by subtracting T_1ρ_ relaxation times measured with different spin lock powers (Fig. 6). Significant differences between different T_1ρ_ relaxation times in infarct and remote areas were not found, and the difference in both areas remained almost constant between the time points (Fig. 6).

Parameters of cardiac function indicate that MI has developed as a function of time (Table 2). Specifically EF decreased as a function of time and, three weeks after MI, EF had decreased to 0.35 (Table 2). Increased cardiac output between days 1 and 7 was found, although it was assumed to decrease (Table 2).

**Table 2:** Values of cardiac function parameters which are presented as mean ± SD

Cardiac function parameters	Day 1	Day 3	Day 7	Day 21
End diastolic volume (mm^3^)	48.4 ± 20.7	47.1 ± 24.6	72.9 ± 26.7	90.3 ± 30.2*,^□^
End systolic volume (mm^3^)	22.7 ± 13.0	21.9 ± 13.8	39.9 ± 17.6	58.0 ± 18.5**,^□□^
Ejection Fraction (%)	0.55 ± 0.12	0.56 ± 0.09	0.46 ± 0.09	0.35 ± 0.04**,^□□^
Cardiac output (mm^3^)	14,570 ± 3680	15,140 ± 5770	20,280 ± 8130*	19,800 ± 8680
Heart rate (bpm)	580.2 ± 82.5	599.0 ± 64.9	615.5 ± 48.0	632.0 ± 52.3

Differences between time points of different cardiac measures were analyzed by One-way ANOVA with Bonferroni post hoc test (*=*P*<0.05 for difference to day 1) (^□^=*P*<0.05, ^□□^=*P*<0.01 for difference to day 3)

## Discussion

In this murine study, relaxation times were measured in infarct and remote areas at several time points after MI. Infarct size was measured based on different relaxation times, the results were compared to infarct sizes derived from CMR LGE-image and Sirius Red-stained histology sections. MI size from T_1ρ_, T_RAFF2_ and T_RAFF4_ relaxation time maps showed a high correlation with MI size determined based on Sirius Red-stained histology sections.

T_RAFF2_ relaxation times in infarct areas increased significantly from day 1 to day 21 after the LAD ligation and the RRTD values increased as a function of time. Differences between infarct and remote areas in T_RAFF2_ relaxation times were statistically significant. Low AOE-value obtained with T_RAFF2_ relaxation time demonstrate that elevated T_RAFF2_ denotes permanently damaged area. These findings together with a high correlation between T_RAFF2_ and histology derived infarct size, demonstrate that T_RAFF2_ relaxation time detects MI area with high accuracy. Previously, T_RAFF2_ relaxation time has been measured in a rat malignant glioma model where T_RAFF2_ showed a high correlation with decreased cell density in tumors [30]. Furthermore, cell density decreases in the infarct area as the tissue is replaced by fibrotic tissue, which leads to an increase of extracellular space [10]. Most likely, increase of T_RAFF2_ in both of these cases is caused by increases in extracellular space and, therefore, an increase in free water content. This suggest that fibrotic tissue can be differentiated healthy tissue using the T_RAFF2_ map. This is important since fibrosis detection also plays a central role in MI detection. Our results demonstrate the potential of T_RAFF2_ mapping to determine and accurately assess the MI area in both acute and chronic phase of the disease. Another explanation for elevated T_RAFF2_ is altered ^1^H chemical exchange between water and macromolecules due to changes induced by infarct in exchange of populations, exchange rates, or chemical shifts between exchanging sites [30]. The extracellular pH may also change in MI, which may induce alterations to exchange rates [30].

Our results showed that T_RAFF4_ relaxation times were elevated at all time points in the MI area and the difference between infarct and remote areas in T_RAFF4_ was significant. Our findings suggest that T_RAFF4_ relaxation time mapping can be applied to detect chronic MI since infarct size derived from T_RAFF4_ map correlated highly (R^2^ = 0.94, *P* < 0.001) with histology derived infarct area together with small AOE-value.

T_1ρ1250_ relaxation time determined at infarct area was significantly higher when compared to remote areas of the myocardium. In addition, infarct size derived from T_1ρ1250_ map correlated with infarct size from Sirius Red sections (R^2^ = 0.96, *P* < 0.01) and its AOE-value was close to zero. The largest RRTD between infarct and remote area was found with T_1ρ625_. Infarct size based on T_1ρ625_ and T_1ρ400_ relaxation time maps showed high correlations (R^2^ = 0.99, *P* < 0.001, R^2^ = 0.96, *P* < 0.05, respectively) with Sirius Red staining but T_1ρ625_ showed the largest AOE-value (15.4 ± 6.1%). T_1ρ_ relaxation times have previously been known to be two times longer in scar tissue than in normal myocardium in porcine heart [16]. However, the origin of T_1ρ_ increase at MI is still unclear. Most likely it is due to increased fibrosis content, cellularity, or ^1^H chemical exchange [11, 31]. In addition, there is a suggestion that leaking protein material from sarcolemma into extracellular space minimizes effects of proteins on water molecules or macromolecules in acute MI in patients and swine [10, 17]. Selective sensitivity to correlation times near to 1/(γB_1_) is an advantage of T_1ρ_ relaxation time [21].

In a previous study, the T_1ρ_ relaxation time with different spin-lock powers increased significantly 7 days after MI compared to the remote area [21] and T_1ρ_ relaxation times elevated almost monotonically during 2 weeks after MI [21]. These findings were explained by granulation and scar tissue formation [11, 21]. Similar increase was detected in this study with T_1ρ_s, T_RAFF2_, and T_RAFF4_.

T_2_ relaxation times increased significantly from day 1 to day 3 in infarct area and T_2_ relaxation times in infarct area were significantly higher than T_2_ relaxation times in remote area. These results are in line with previous findings showing that damaged area in myocardium is reversible in acute phase of MI [12, 18, 32]. It has previously been shown that T_2_ relaxation time overestimates the size of acute MI compared to chronic MI, since the inflammation and edema have resolved from the chronic MI [18]. Similar findings were observed in this study since infarct percentages based on T_2_ maps were higher as compared to percentages based on the other relaxation times at early time points. Therefore, increased T_2_ relaxation time shows the area of edema rather than the actual size of MI [18]. T_2_ relaxation times did not differ between day 1 and day 21, which suggests that part of the damage in myocardium was reversible without the scar formation. In a previous study, T_2_ relaxation times were significantly higher in acute MI area compared to chronic MI area [12].

The MI areas defined based on CMR images were in good agreement with the areas determined with Sirius Red-stained histology. To our knowledge, elevated relaxation times, at least T_1ρ_ relaxation times, at 21 days after MI are due to fibrosis and scar tissue formation. The locations of fibrotic areas in Sirius Red-stained sections and the elevated relaxation times on relaxation time maps agreed well. Notable, our results are based on quantitative relaxation times instead of weighted images. These results also suggest that especially T_RAFF2_, T_RAFF4_ and T_1ρ_ relaxation time mappings may be useful for broad range of clinical applications where myocardial tissue characterization is needed, for example in myocarditis, various size and locations of scars, sarcoidosis and hypertrophy. Operating at lower main magnetic field strengths will have some impact on rotating frame relaxation times; however, the main influence is the strength of the spin-lock power [33]. Water molecules with correlation times close to 1/(γB_1_) contribute to rotating frame contrasts [33]. Adaption of rotating frame measurements to MI characterization at clinics needs further study since we demonstrated advantages of relaxation times only in MI mouse model where one artery (LAD) was occluded.

MI area was detectable in all relaxation time maps and it was the most visible at day 21. At the first time points, the MI area was larger than at the later time points most likely due to inflammation surrounding the infarction area. In a mouse model, MI area consists of over 90% of necrotic tissue only two days after LAD ligation and the necrotic tissue is transformed into granulation tissue one week after MI and into scar two weeks after the LAD ligation [5].

The LAD ligations were successfully performed since our results show a clear visibility of infarct in Sirius Red-stained sections and decreased EF values as a function of time. Additionally, EDV and ESV tend to increase post MI due to changes in physiology of the myocardium. Changes in physiology with decreased perfusion inside the myocardium lead also to a decrease of EF as a function of time. Our EF values agreed with EF values reported in the literature [34, 35]. Increased EDV and ESV at day 21 compared to day 1 after MI resulted in an increased stroke volume (23%) which together with increased heart rate (8%) lead to increased cardiac output. The ventricular dilation after MI is typical [34, 35] and was also observed in our cine images. However, increased stroke volume after MI is rare [34, 35]. Differences in anesthesia level or mice increased tolerance to anesthetic during subsequent CMR exams might be the reasons for the increased heart rates.

## Conclusions

All relaxation time maps showed high contrast between infarct and remote areas. T_1ρ_, T_RAFF2_ and T_RAFF4_ relaxation time maps correlated significantly with the infarct size determined by histology. As a conclusion, T_RAFF2_ and T_RAFF4_ relaxation time maps can be used to accurately determine infarct size in mouse myocardial infarct without the use of contrast agent with clinically tolerable specific absorption rates.

## Abbreviations

AHP: Adiabatic half passage
AOE: Amount of overestimation
CMR: Cardiovascular magnetic resonance
CO: Cardiac output
ECG: Electrocardiogram
EDV: End-diastolic volume
EF: Ejection fraction
ESV: End-systolic volume
Gd: Gadolinium
LAD: Left anterior descending coronary artery
LGE: Late gadolinium enhancement
MI: Myocardial infarction
RAFF: Relaxation along a fictitious field
RF: Radiofrequency
ROI: Region of interest
RRTD: Relative relaxation time difference
SAR: Specific absorption rate
TSL: Time to spin lock
